# Identification of immunotherapy biomarkers for improving the clinical outcome of homologous recombination deficiency patients with lung adenocarcinoma

**DOI:** 10.18632/aging.204957

**Published:** 2023-08-11

**Authors:** Xiang Zhou, Rongjian Xu, Tong Lu, Ran Xu, Chenghao Wang, Bo Peng, Xiaoyan Chang, Zhiping Shen, Kaiyu Wang, Jiaxin Shi, Jiaying Zhao, Lin-You Zhang

**Affiliations:** 1 Department of Thoracic Surgery, The Second Affiliated Hospital of Harbin Medical University, Harbin, Heilongjiang 150084, China; 2 Department of Thoracic Surgery, The Affiliated Hospital of Qingdao University, Qingdao, Shandong 266005, China

**Keywords:** HRD, MS4A6A, immunotherapy, lung adenocarcinoma, single-cell RNA sequencing

## Abstract

Homologous recombination deficiency (HRD) is a common molecular signature of genomic instability and has been shown to be a biomarker for targeted therapies. However, there is a lack of studies on the role of HRD changes in lung adenocarcinoma (LUAD) transcriptomics. HRD scores were determined using single nucleotide polymorphism (SNP) array data from LUAD patients from The Cancer Genome Atlas (TCGA) database. Transcriptional data from patients with different scores were analyzed to identify biomarkers associated with HRD. Candidate biomarkers were validated using Gene Expression Omnibus (GEO)-sourced datasets and an immunotherapy cohort. According to the bulk transcriptome and clinical characteristics of 912 LUAD patients and Single-cell RNA-seq of 9 LUAD patients from TCGA and GEO databases, we observed increased MS4A6A expression in HRD tumors; high MS4A6A expression predicted improved survival outcomes. Furthermore, a comprehensive analysis of the tumor immune microenvironment (TIME) revealed a positive correlation between *MS4A6A* expression and neoantigen loading and immune cell infiltration. Additionally, the immunotherapy cohort confirmed the possibility of using *MS4A6A* as a biomarker. Collectively, we suggest that *MS4A6A* is associated with HRD and provide a new perspective toward identifying promising biomarkers for immunotherapy.

## INTRODUCTION

In recent years, tumor immunotherapy has brought about revolutionary changes in cancer treatment. Lung cancer poses one of the greatest problems in antitumor treatment, and according to cancer statistics, lung cancer ranks first in terms of incidence and mortality [1]. The emergence of immunotherapy has significantly changed the landscape of lung cancer treatments. Although immunotherapy has made breakthroughs, the objective remission rate (ORR) in NSCLC, without varying treatment populations, is approximately 20% [2, 3]. The ORR remains below 50% even in populations with more than 50% programmed death-ligand 1 (PD-L1) expression [4, 5]. Thus, effective biomarkers are essential for selecting immunotherapy populations and improving the efficacy of immunotherapy.

Based on previous studies, immunotherapy-related markers can be broadly classified into the following four categories: 1) tumor cell-related biomarkers, such as PD-1, PD-L1 expression, tumor mutational burden (TMB), DNA damage response (DDR) pathway, and neoantigens; 2) tumor microenvironment (TME)-related markers, such as tumor-infiltrating immune cells (CD4^+^ and CD8^+^ T cells); 3) liquid biopsy markers, such as peripheral blood cells and circulating tumor DNA; and 4) host-related biomarkers, such as intestinal symbionts and host germline genetic characteristics. Homologous recombination deficiency (HRD) usually refers to a state of DNA repair dysfunction at the cellular level, which can be caused by many factors, such as germline or somatic mutations in homologous recombination repair (HRR)-related genes and epigenetic inactivation. HRD can affect the DDR pathway by introducing insertions/deletions in nucleic acid sequences, copy number abnormalities, and chromosomal cross-linking, resulting in genomic and chromosomal instability [6]. HRD is present in various malignancies, particularly ovarian and breast cancers [7, 8]. The status and extent of HRD have emerged as novel biomarkers for the clinical application of PARP inhibitors in patients with advanced ovarian cancer [9–11]. In a previous study, Kadouri et al. observed that HRD is a risk factor affecting the prognosis of lung adenocarcinoma (LUAD) [12]. However, few studies have reported on HRD and LUAD, and the specific mechanism by which HRD affects the prognosis of LUAD remains unclear.

**Figure 1:**
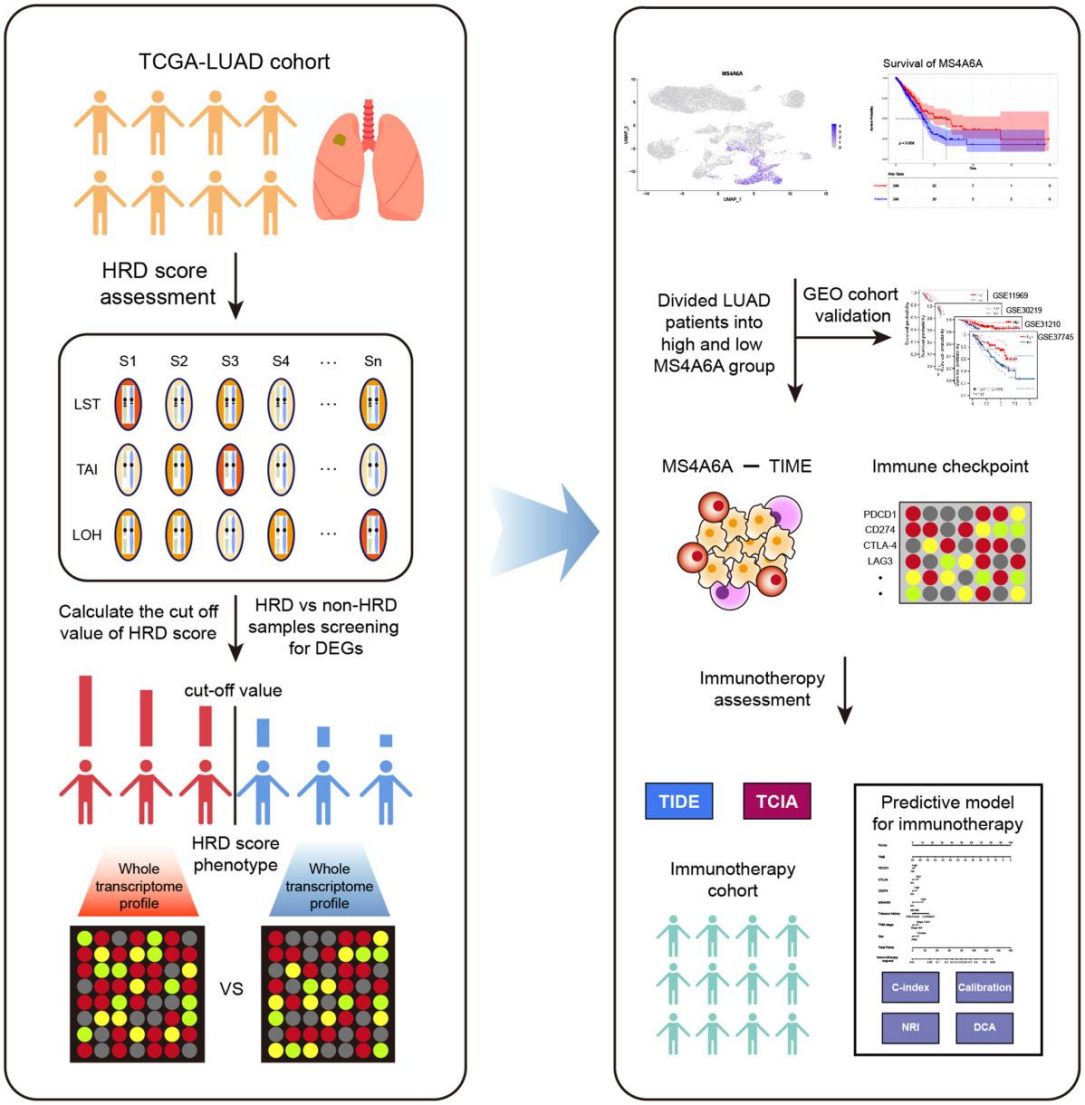
**Workflow of this study.** The analysis process of HRD-related RNA is shown on the left. HRD scores were obtained by calculating LOH, LST, and TAI for each sample of TCGA-LUAD. Patients were classified as HRD and non-HRD according to cut-off values. The screening and identification of markers are shown to the right. *MS4A6A* was identified as a potential immunotherapeutic marker by survival analysis, immune microenvironment, and immune checkpoint analysis.

To explore the association between HRD-induced genomic instability and immunotherapy biomarkers in LUAD patients, we extracted transcriptomic data and mutation data from The Cancer Genome Atlas (TCGA) database. We calculated HRD scores for each LUAD patient. By analyzing the differences in transcriptome levels between the HRD and non-HRD groups, we observed elevated *MS4A6A* expression in HRD patients and that these patients had a better prognosis. Four datasets from the Gene Expression Omnibus (GEO) database were used to validate our results. Furthermore, we revealed that *MS4A6A* expression was positively correlated with multiple infiltrated immune cells in the TME, such as CD4+ T cells, CD8+ T cells, as well as immune checkpoints (ICPs), such as PD-1 and PD-L1. IMvigor210, an immunotherapy cohort, suggested that *MS4A6A* was a better predictor than PD-1, PD-L1, or CTLA-4. Therefore, the findings of this study provide possible directions for immunotherapy biomarkers and are valuable for understanding the relationship between genomic instability and TME in LUAD patients and improving clinical outcomes in patients undergoing immunotherapy (Figure 1).

## MATERIALS AND METHODS

### Data collection and pre-processing

The data used in the current study are accessible from publicly available databases. Transcriptomic data and SNP, as well as the corresponding clinical characteristics and follow-up information (*n* = 485) of LUAD, were obtained from the TCGA database. To analyze the transcriptomic data, count values and transcripts per kilobase of exon model per million mapped read (TPM) values were extracted. The “Masked Somatic Mutation” was selected as the somatic mutation data. The maftools R package [13] was used to visualize the somatic mutation landscape and calculate the TMB for each LUAD patient. Partial genomic alteration scores (percentage of chromosomal copy number altered regions outside the measured regions) and MSI-Sensor scores were obtained from the cBioPortal database (http://www.cbioportal.org). Detailed information is provided in Table 1.

**Table 1:** TCGA-LUAD clinical baseline information.

**Characteristic**	**Non-HRD (*n* = 137)**	**HRD (*n* = 348)**
Sex, *n* (%)		
Female	74 (15.4%)	183 (38.1%)
Male	60 (12.5%)	163 (34%)
Stage, *n* (%)		
Stage I	79 (16.5%)	184 (38.4%)
Stage II	27 (5.6%)	88 (18.4%)
Stage III	21 (4.4%)	55 (11.5%)
Stage IV	6 (1.3%)	19 (4%)
Age, median (IQR)	69 (61, 74)	65 (58, 72)
MSIsensor Score, median (IQR)	0 (0, 0.06)	0.02 (0, 0.13)
Mutation Count, median (IQR)	99.5 (40, 188.75)	254 (122, 451.5)
Fraction Genome Altered, median (IQR)	0.12 (0.04, 0.22)	0.31 (0.17, 0.45)
TMB (nonsynonymous), median (IQR)	3.3 (1.52, 7.13)	8.73 (4.08, 16.31)

Datasets containing LUAD samples and clinical follow-up information were retrieved from the GEO database, and four datasets were included: GSE11969 [14], GSE30219 [15], GSE31210 [16], and GSE37745 [17]. The scRNA-seq data were obtained from GSE189357 [18] which includes 9 LUAD samples from nine resected samples of treatment-naïve patients. The array information of these datasets is shown in Supplementary Table 1. Additionally, the IMvigor210 [19] (Table 2) and GSE126044 [20] cohorts, which contained data on immunotherapy, were included in this study. Each dataset from the GEO database sources was normalized and annotated with an ID based on platform information.

**Table 2:** IMvigor210 clinical baseline information.

**Characteristic**	**MS4A6A-low (*n* = 174)**	**MS4A6A-high (*n* = 174)**
Bir Response, *n* (%)		
CR/PR	35 (11.7%)	33 (11.1%)
SD/PD	118 (39.6%)	112 (37.6%)
Sex, *n* (%)		
Female	39 (11.2%)	37 (10.6%)
Male	135 (38.8%)	137 (39.4%)
Immune phenotype, *n* (%)		
Desert	56 (19.7%)	20 (7%)
Excluded	67 (23.6%)	67 (23.6%)
Inflamed	22 (7.7%)	52 (18.3%)
TMB, median (IQR)	0.9 (0.53, 1.43)	0.94 (0.43, 1.87)

### Calculation of HRD and neoantigen scores

The HRD score was defined as the unweighted sum of the loss of heterogeneity (LOH) [21], telomere allelic imbalance (TAI) [22], and massive state transition (LOS) scores [23, 24]. The neoantigen load, that is, the number of peptides predicted to bind to major histocompatibility complex (MHC) proteins, was determined based on the HLA type derived from RNA sequencing data. The neoantigen load is expressed as single nucleotide variants (SNV) and insertion and deletion (indel) mutations. The values of HRD, neoantigen load, and mutation rate (number of single-nucleotide mutations) were compiled from a pan-cancer mapping study by Thorsson et al. [25]. The detailed information is provided in Supplementary Tables 2, 3.

### Identification of independent prognostic genes associated with HRD score

We used the DESeq2 R package [26] for differential analysis of the HRD and non-HRD groups, filtering out low-expression genes and selecting |log2(fold change)| > 1.5, adj. *P* < 0.05 as the criteria. log2(fold change) > 1.5 were considered as highly expressed genes and < −1.5 for lowly expressed genes in HRD. Volcano plots were used to visualize differentially expressed genes (DEGs).

Univariate Cox regression analysis of the differentially expressed genes was performed using the survival R package (https://CRAN.R-project.org/package=survival), and a *P*-value < 0.05 was selected as the cut-off value. The screened genes were subsequently analyzed by least absolute shrinkage and selection operator (LASSO) regression to determine the maximum prediction accuracy and the balance between minimizing explanatory accuracy [27]. Finally, independent prognostic factors were determined using a multivariate Cox regression analysis. A *P*-value < 0.05 was considered an independent prognostic factor, HR >1 as a risk factor, and HR <1 as a protective factor.

### Selection of immunotherapy biomarkers by scRNA-seq analysis

An analysis of 10x scRNA-seq data was conducted by R packages, including “Seurat” [28] and “SingleR” [29] we utilized the “Seurat” R package to preprocess and analyze single-cell RNA sequencing (scRNA-seq) data. The scRNA-seq data were normalized using the “NormalizeData” function of the “Seurat” package, with the normalization method set as “LogNormalize”. The resulting normalized data were then converted into a Seurat Object. The percentage of mitochondrial or ribosomal genes was calculated and low-quality cells were excluded to ensure the quality control (QC) [30]. We excluded samples with gene counts below 200 or above 3000, as well as those with a ribosomal RNA proportion exceeding 20%. Then, the top 3,000 genes with high variability were identified using the “FindVariableFeatures” function. To reduce the dimensionality of the scRNA-seq data, we performed principal component analysis (PCA) using the “RunPCA” function of the “Seurat” R package, based on the top 3,000 variable genes. Significant principal components (PCs) were identified through JackStraw analysis, and we selected the first 15 PCs for cell clustering analysis according to the proportion of variance explained. For cell clustering analysis, we utilized the “FindNeighbors” and “FindClusters” functions in the “Seurat” package. A k-nearest neighbor graph was constructed based on Euclidean distance in PCA using “FindNeighbors” to determine the closest neighbors of each cell. Cells were visualized using uniform manifold approximation and projection (UMAP) dimensionality reduction techniques for cell classification. To identify differentially expressed genes (DEGs) for each cluster, we used the “FindAllMarkers” function in the “Seurat” package, following Wilcoxon-Mann-Whitney tests. Marker genes for each cluster were identified using adjusted *p*-value < 0.01 and |log2 (fold change)| >1 as threshold values. We conducted a manual annotation, as described in the study by Maynard et al. [31], to identify and classify different cell types in our experimental samples. Finally, FeaturePlot and vlnPlot functions embedded in the “seurat” package were applied to visualize the cellular distribution of independent prognostic genes in the scRNA-seq dataset.

### Gene set enrichment analysis

LUAD patients were divided into high and low *MS4A6A* groups according to the median *MS4A6A* expression value, and differential analysis was performed using the DESeq2 package. Gene Set Enrichment Analysis (GSEA) was performed using the clusterProfiler R package [32] to calculate the normalized enrichment score (NES) for each gene set and identify the signaling pathways enriched in the high- and low-*MS4A6A* expression groups. The selected gene set was selected as “c2.cp.v7.2. symbols” and false discovery rate (FDR) <0.25 was selected as a cut-off value.

### Immune cell infiltration analysis

The microenvironmental characteristics of the tumors were assessed using the ESTIMATE R package [33]. ESTIMATE analysis quantifies immune activity (level of immune infiltration) in the tumor microenvironment based on its gene expression profile to obtain an immune score for each sample.

To investigate the abundance of immune cell infiltration in bulk tumor tissues, we used the Tumor Immune Estimation Resource (TIMER) database (https://cistrome.shinyapps.io/timer/) to predict the relative abundance of six types of infiltrating immune cells, including macrophages, dendritic cells, B cells, T cells, and neutrophils. Additionally, we extracted the expression of antigen-presentation-related genes, including those encoding MHC class I/II (I: *HLA-A*, *HLA-B*, and *HLA-C*; II: *HLA-DP*, *HLA-DM*, *HLA-DOA*, *HLA-DOB*, *HLA-DQ*, and *HLA-DR*) and antigen-binding molecules such as *B2M* and *TAP1/2*, and performed a correlation analysis between the expression of these molecules and *MS4A6A*.

### Assessment of immunotherapy

Tumor immune dysfunction and exclusion (http://tide.dfci.harvard.edu/) can characterize T cell dysfunction by calculating tumor immune dysfunction and exclusion (TIDE) scores and evaluating the interaction of gene expression with the level of cytotoxic T lymphocytes (CTL) infiltration, which evaluates patient survival and response to immunotherapy [34]. Therefore, we assessed the clinical response to immunotherapy in patients with high and low *MS4A6A* expression by calculating the TIDE scores in LUAD patients. The Cancer Immunome Atlas (TCIA) was developed and maintained by the Institute of Bioinformatics [35]. This database allows querying the gene expression of specific immune-related genomes, cellular composition of immune infiltrates (characterized by genomic enrichment analysis and deconvolution), neoantigens, cancer-germline antigens, and immunophenotype scores. Therefore, we assessed the potential immunotherapeutic effects of high and low *MS4A6A* expression levels by extracting immunophenotype scores.

In the investigation of the IMvigor210 and GSE126044 cohorts, we assessed the accuracy of *MS4A6A* against common immunotherapy-related markers, including TMB, *PD-1*, *PD-L1*, and *CTLA4*. We constructed a clinical prediction model that evaluated the effect of response to immunotherapy using logistic regression. Bootstrapping was used with 1000 iterations for re-sampling. A calibration curve was used to measure the consistency of the model. An integrated discrimination improvement (IDI) curve was used to assess its improvement, and decision curve analysis (DCA) was used to measure its clinical effect.

### Statistics analysis

Data processing and analysis were performed using the R software (version 4.0.2). The statistical significance of normally distributed variables was estimated by independent Student’s *t*-tests, whereas the differences between two groups of variables with non-normal distribution were assessed using the Mann–Whitney *U*-test (i.e., Wilcoxon rank-sum test). The chi-square and Fisher’s exact tests were used to assess the statistical significance between the two groups of categorical variables. Kaplan–Meier (KM) survival curves were used to compare the survival rates of patients in the two groups. The log-rank test (log-rank test) was used to evaluate the significance of survival time differences between the two groups. LASSO analysis was performed using the glmnet R package. Nomograms and calibration curves were constructed using the rms package (https://CRAN.R-project.org/package=rms), and DCA was plotted using the rmda package. All statistical *P*-values were two-sided, and statistical significance was set at *P* < 0.05.

### Data availability statement

The datasets and source codes used or analyzed during the current study are available from the corresponding author upon reasonable request.

## RESULTS

### HRD score is significantly associated with prognosis and mutational characteristics in LUAD patients

**Figure 2:**
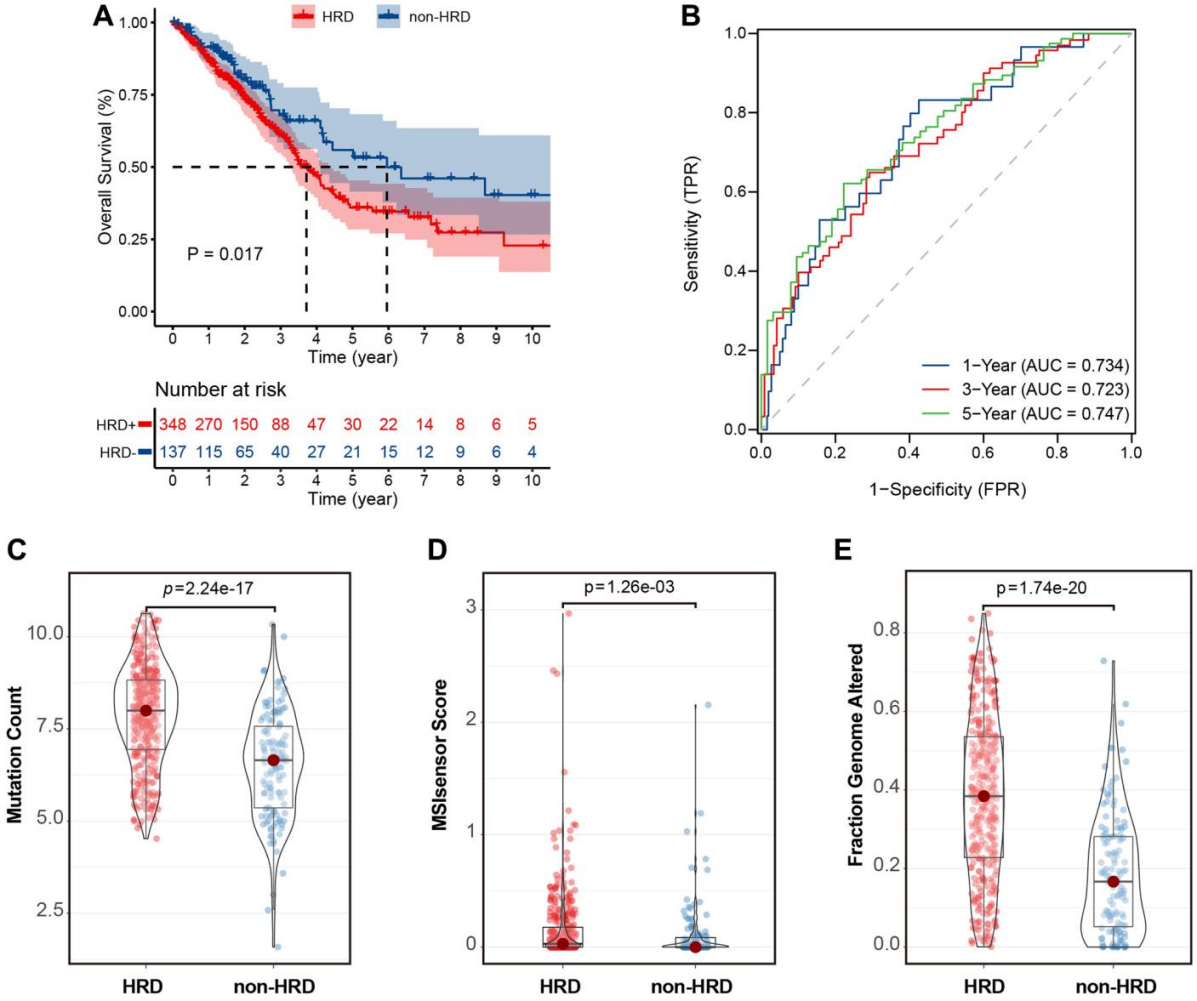
**HRD scores were significantly associated with prognosis and mutation characteristics in the TCGA-LUAD cohort.** (**A**) KM curve of overall survival of patients with HRD or non-HRD tumors in the TCGA-OSC cohort. (**B**) ROC curves of HRD scores in the TCGA-LUAD cohort. (**C**) Violin plots of somatic mutations in the HRD and non-HRD groups. Somatic mutation counts were significantly higher in the HRD group than in the non-HRD group. (**D**) Violin plots of MSI-Sensor in HRD and non-HRD groups. MSI-Sensor in the HRD group were significantly higher than those in the non-HRD group. (**E**) Violin plots of genomic alterations in the HRD group and non-HRD group. ^*^P < 0.05, ^**^P < 0.01, ^***^P < 0.001.

The HRD scores were calculated based on the LOU, LST, and TAI scores in the TCGA-LUAD dataset. The optimal cut-off value of the HRD score was determined by calculating the minimum *P*-value in the log-rank test. Patients with HRD scores >15 were considered to belong to the HRD group, whereas those with HRD scores ≤15 were considered to belong to the non-HRD group. The KM curve showed that the overall survival (OS) of patients in the non-HRD group was much longer than that of patients in the HRD group (log-rank test, *P* = 0.017) (Figure 2A). The 1-, 3-, and 5-year ROC curves of survival were plotted. Their AUCs were estimated to be 0.734, 0.723, and 0.747, respectively (Figure 2B), indicating that the survival between HRD and non-HRD patients at 1, 3, and 5 years was significantly different and that the HRD score may serve as a potential prognostic biomarker. Subsequently, we investigated the relationship between HRD scores and other genomic instability features, such as MSI-sensor, genomic alteration fractions, and somatic mutation counts. The results revealed that the median somatic cumulative mutation count was significantly higher in the HRD group than in the non-HRD group (Wilcoxon signed-rank test, *P* < 0.001; Figure 2C) and was higher in the HRD group than in the non-HRD group (Wilcoxon signed-rank test, *P* < 0.001; Figure 2D). The fraction genome altered was also higher in the HRD group (Wilcoxon signed-rank test, *P* < 0.0001; Figure 2E). These findings indicate that patients with HRD have significantly higher genomic instability than those without HRD.

### Differential mutation landscapes in HRD and non-HRD groups

**Figure 3:**
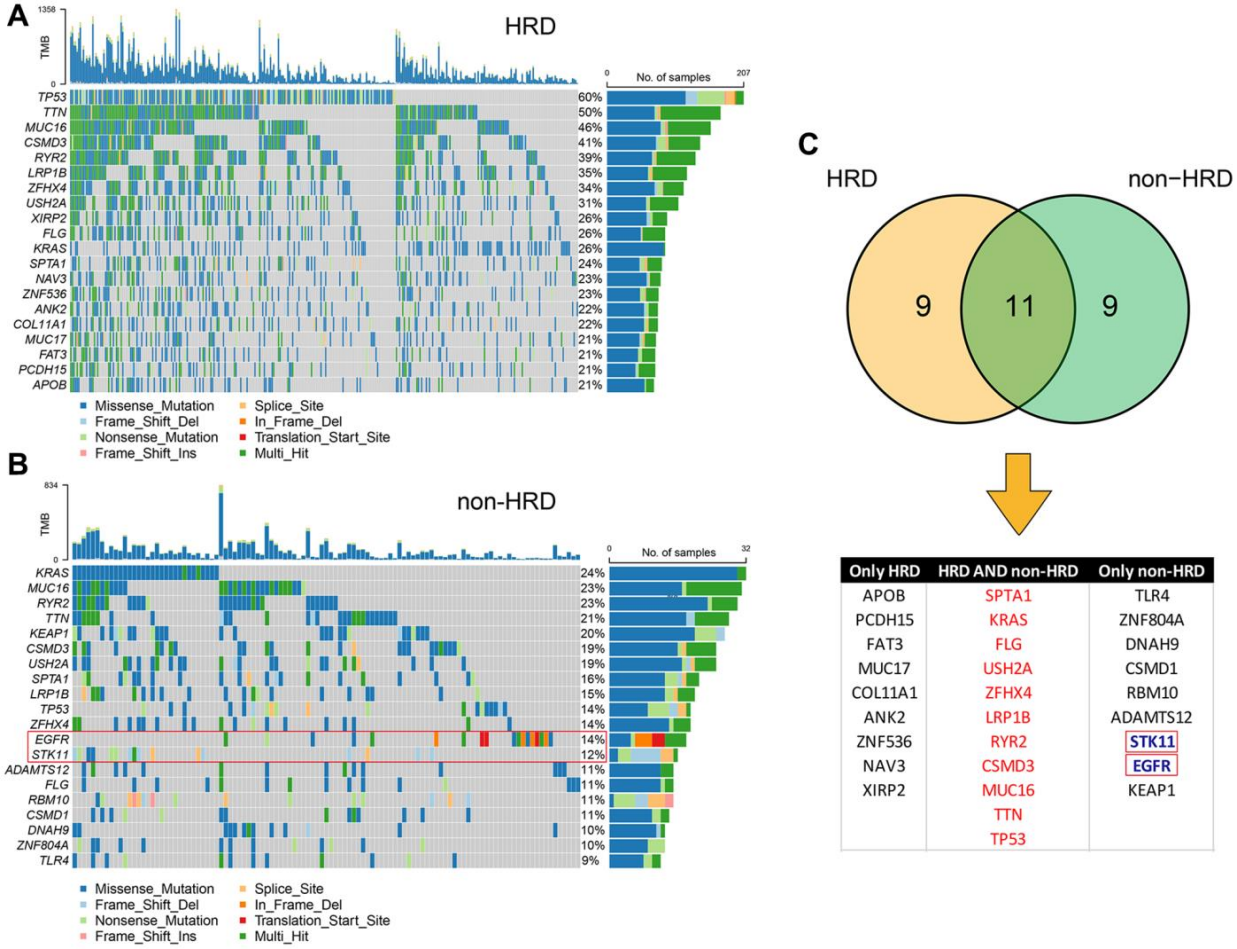
**Mutational landscape of HRD and non-HRD patients.** (**A**) Top 20 mutation landscape of HRD patients in the TCGA-LUAD cohort. (**B**) Top 20 mutation landscape of non-HRD patients in the TCGA-LUAD cohort. The genes in red boxes are actionable genes. (**C**) Overlapping information of HRD and non-HRD mutated genes; actionable genes are only in non-HRD.

Genomic features, such as oncogene activation (e.g., *ERBB2* amplification, *EGFR* tyrosine kinase mutations) and tumor suppressor gene inactivation (e.g., *MMR*, *BRCA1*/2), have been shown to strongly correlate with the clinical response to targeted therapies. Therefore, we compared the mutation landscape between the HRD and non-HRD groups. (Figure 3A, 3B) The results showed that the mutation landscape of the non-HRD group was significantly different from that of the HRD group. Only 11 of the top 20 genes with the highest mutation rates in the two groups overlapped (Figure 3C), and the mutation frequencies of the overlapping genes also differed significantly. For example, the *TP53* mutation frequency was 60% and 14% in the HRD and non-HRD groups, respectively. Furthermore, by screening actionable genes in the OncoKB database (https://www.oncokb.org/actionableGenes), two of the 20 genes with the highest mutation frequency in the non-HRD group were identified as biomarkers for targeted drugs (*STK11* and *EGFR*). The mutation frequencies of these two genes in the non-HRD group were 14% and 12%, respectively. These results showed that HRD and non-HRD patients had different mutated genes. Non-HRD patients had actionable genes and drug targets suitable for targeted therapy. It further supported HRD as a potential biomarker for LUAD.

### *MS4A6A* gene expression is positively correlated with HRD score and is an independent prognostic factor in LUAD patients

**Figure 4:**
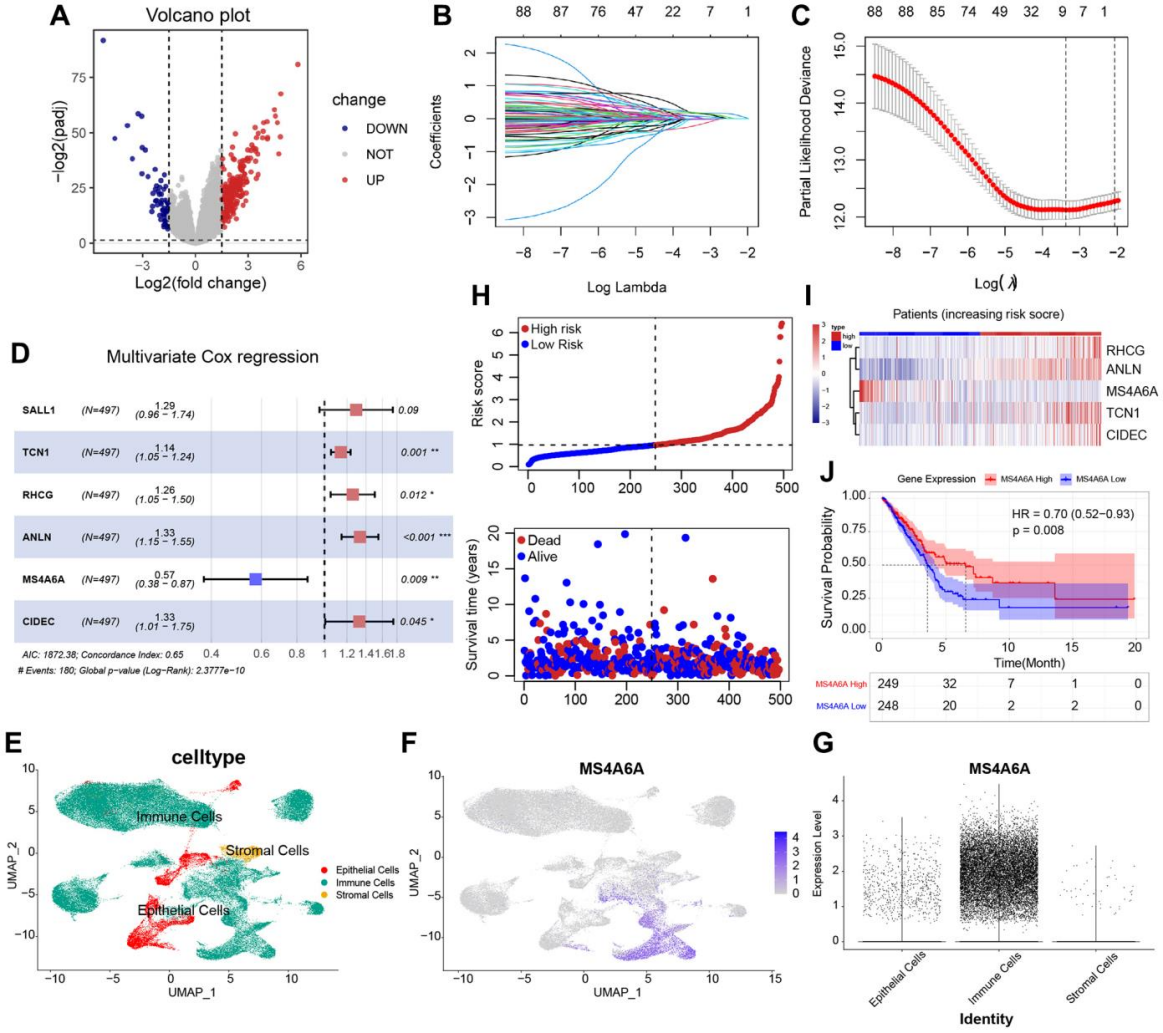
**Identification of HRD-related prognostic RNA.** (**A**) Differential analysis based on HRD vs. non-HRD patients, 326 DEGs were obtained, of which 266 genes were expressed up in HRD patients, and 60 genes were expressed down in HRD patients. (**B**, **C**) After univariate Cox regression screening, 89 prognostic genes were obtained and analyzed by LASSO regularized dimensionality reduction and eliminated the covariance between variables, after which nine genes were obtained. (**D**) Multivariate Cox regression screening of independent prognostic genes, containing five genes, of which all were risk factors except for *MS4A6A*. (**E**) Cellular distribution of 109649 cells clustered into 3 unique subsets among all merged lung adenocarcinoma tissue samples. (**F**) FeaturePlot depicting the distribution of MS4A6A. (**G**) vlnPlot showing the expression levels of MS4A6A in different cell subsets. (**H**) Risk plots of multifactorial Cox regression with the distribution of risk score in the upper layer, the distribution range of survival information in the lower layer. (**I**) The expression characteristics of five genes in high and low-risk group. (**J**) KM curves of *MS4A6A* (log-rank test).

To identify mRNAs associated with HRD scores, we performed a differential analysis of RNA-seq data from HRD and non-HRD patients. A total of 326 DEGs were identified, of which 266 genes were highly expressed, and 60 genes were lowly expressed in the HRD group (Figure 4A). To identify DEGs associated with patient HRD scores and prognosis, we then conducted a univariate Cox regression analysis of the TCGA-LUAD cohort, including 326 DEGs. Univariate analysis and log-rank tests were used to identify 89 genes with prognostic potential (*P* < 0.05). LASSO-Cox proportional risk regression and 10-fold cross-validation were performed on these prognosis-related genes to screen for independent prognostic factors. LASSO coefficient profiles were generated for log lambda and the optimal λ value corresponding to the eight variables (Figure 4B, 4C). After performing stepwise multivariate Cox regression analysis, *SALL1*, *TCN1*, *RHCG*, *ANLN*, *MS4A6A*, and CIDEC were identified as independent prognostic factors. In contrast, only *MS4A6A* was a protective factor, and the rest were risk factors (Figure 4D). Then we performed scRNA-seq analysis with GEO dataset: GSE189357 (Supplementary Figure 1A–1D). A total of 109,649 cells were analyzed and distinguished into epithelial cells, immune cells, and stromal cells after QC. (Figure 4E). FeaturePlot (Figure 4F) and vlnPlot (Figure 4G) visualization indicated MS4A6A has higher expression than other five independent prognostic signatures (Supplementary Figure 1E) in all kinds of cells, especially in immune cells. The assessment of immune cell signatures within the microenvironment of tumors provides crucial insights into the nature and magnitude of immune response in individual cancer patients, as well as their likelihood of responsiveness to immunotherapy [36]. Thus, focusing on immune cell signatures is more suitable for assessing the potential of immunotherapy in cancer and predicting which patients are likely to benefit from this treatment. As shown in risk plots of multifactorial Cox regression with the distribution of risk score, we divided the patients into high risk subgroup and low risk subgroup and survival status indicated that high risk score subgroup owns worse survival outcome (Figure 4H). The expression characteristics of five genes in high and low-risk groups corresponds to the stepwise multivariate Cox regression analysis (Figure 4I). The KM curve of MS4A6A demonstrated that patients with high MS4A6A expression had a better prognosis (*P* = 0.008) (Figure 4J). To investigate whether the *MS4A6A* expression profile has a similar prognostic value in different datasets, we independently confirmed our findings in four datasets from the GEO database: GSE11969, GSE30219, GSE31210, and GSE37745. KM analysis also showed that patients with high *MS4A6A* expression had a better prognosis than those with low *MS4A6A* expression (Supplementary Figure 2A).

### *MS4A6A* expression is positively correlated with TIME

**Figure 5:**
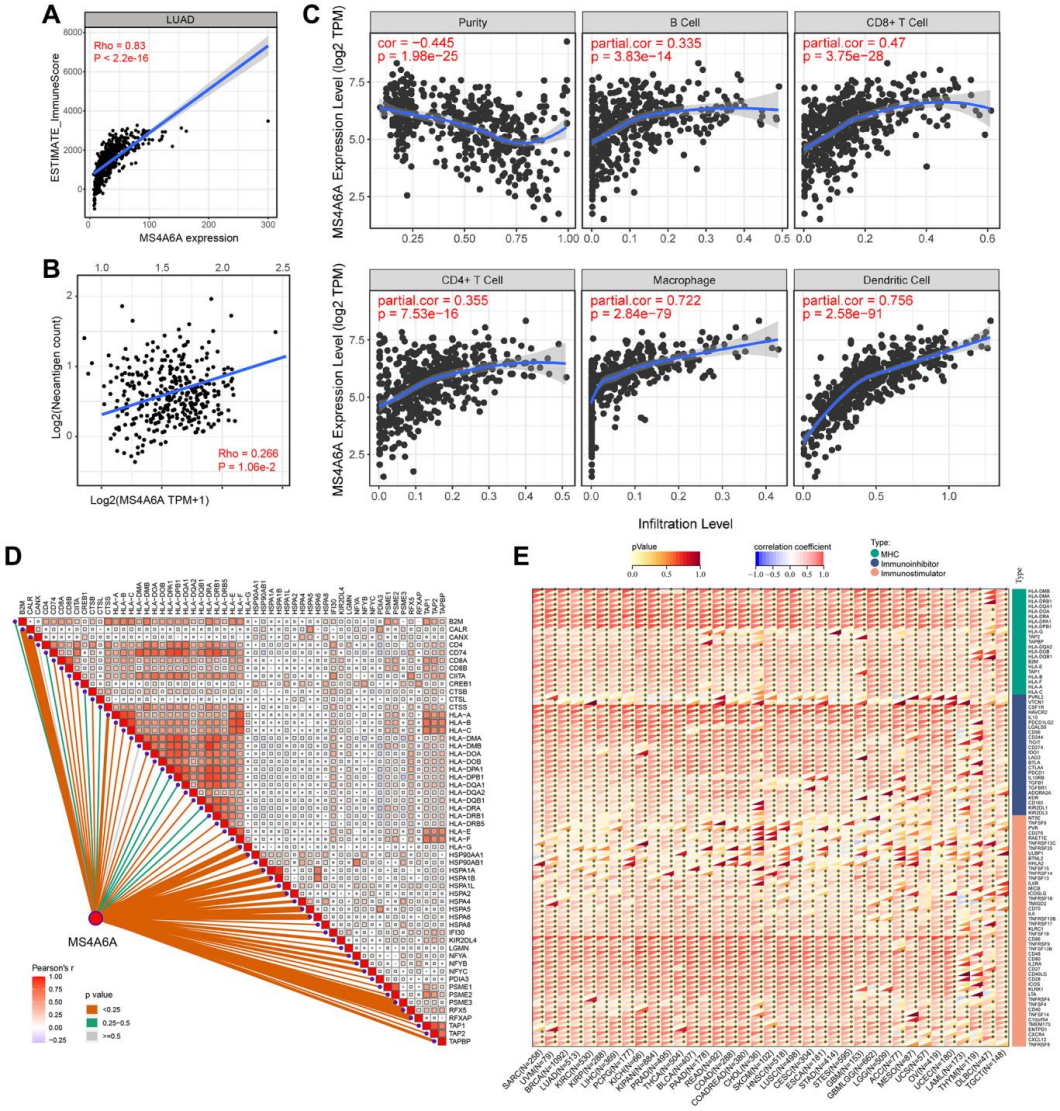
**Relationship between *MS4A6A* expression pattern and TIME.** (**A**) *MS4A6A* expression is positively correlated with ImmuneScore (Spearman rank correlation coefficient, R = 0.83, *P* ≈ 0). (**B**) MS4A6A expression is positively correlated with neoantigen load (Spearman rank correlation coefficient; *P* < 0.05). (**C**) *MS4A6A* expression was positively correlated with immune cell subpopulation (Spearman rank correlation coefficient; *P* < 0.0001). (**D**) *MS4A6A* expression is positively correlated with antigen-related genes. (**E**) Correlation of *MS4A6A* expression signature with antigen-related genes in the TCGA pan-cancer cohort.

High *MS4A6A* expression is associated with a better prognosis than low *MS4A6A* expression. Therefore, we examined the relationship between high *MS4A6A* expression and the tumor microenvironment. To investigate the relationship between MS4A6A expression and immune cell infiltration in the tumor microenvironment, we first calculated the immune score of the TIME in patients with LUAD using the ESTIMATE algorithm. We analyzed the correlation between the immune score and *MS4A6A*. As shown in Figure 5A, *MS4A6A* showed a significant positive correlation with immune score (Spearman’s rank correlation coefficient, Rho = 0.83, *P* < 0.001). Accordingly, we calculated the immune score for each tumor patient in the four GEO datasets and categorized the patients according to the median value of immune infiltration. We found that *MS4A6A* expression was significantly higher in the high immune infiltration group (Supplementary Figure 2B). Furthermore, the predicted neoantigen load was positively correlated with *MS4A6A* expression (Spearman’s rank correlation coefficient, Rho = 0.266, *P* < 0.05) (Figure 5B). We used the TIMER algorithm to estimate the correlation between *MS4A6A* expression and the five types of immune cells to better understand how *MS4A6A* is related to immune cell infiltration. As shown in the scatter plot, *MS4A6A* was significantly positively correlated with macrophages (Rho = 0.722, *P* < 0.001) and dendritic cells (Rho = 0.756, *P* < 0.001), suggesting that *MS4A6A* plays a vital role in antigen presentation and processing. In addition, we found that MS4A6A expression also correlated significantly with CD8+ and CD4+ T cells, suggesting the relevance of *MS4A6A* in tumor killing (Figure 5C). To further investigate the relationship between *MS4A6A* and antigen-presentation-related genes, we analyzed the association between *MS4A6A* expression and MHC class I/II (I: *HLA-A*, *HLA-B*, *HLA-C*; II: *HLA-DP*, *HLA-DM*, *HLA-DOA*, *HLA-DOB*, *HLA-DQ*, *HLA-DR*) and key antigen-binding molecules (such as *B2M*, *TAP1/2*), and observed a significant positive correlation between them (Figure 5D). Notably, we observed that the *MS4A6A* gene was positively correlated with these immune-related genes in LUAD as well as in the other 32 cancers (Figure 5E). *MS4A6A* might be expressed more frequently on the surface of antigen-presenting cells in the tumor microenvironment.

In addition, GSEA analysis of gene expression profiles of the *MS4A6A*-positive and -negative groups revealed that the *MS4A6A* positive group was significantly enriched in DNA repair, DNA mismatch repair, and immune system-related pathways (Supplementary Figure 3).

### *MS4A6A* expression is positively correlated with ICPs

**Figure 6:**
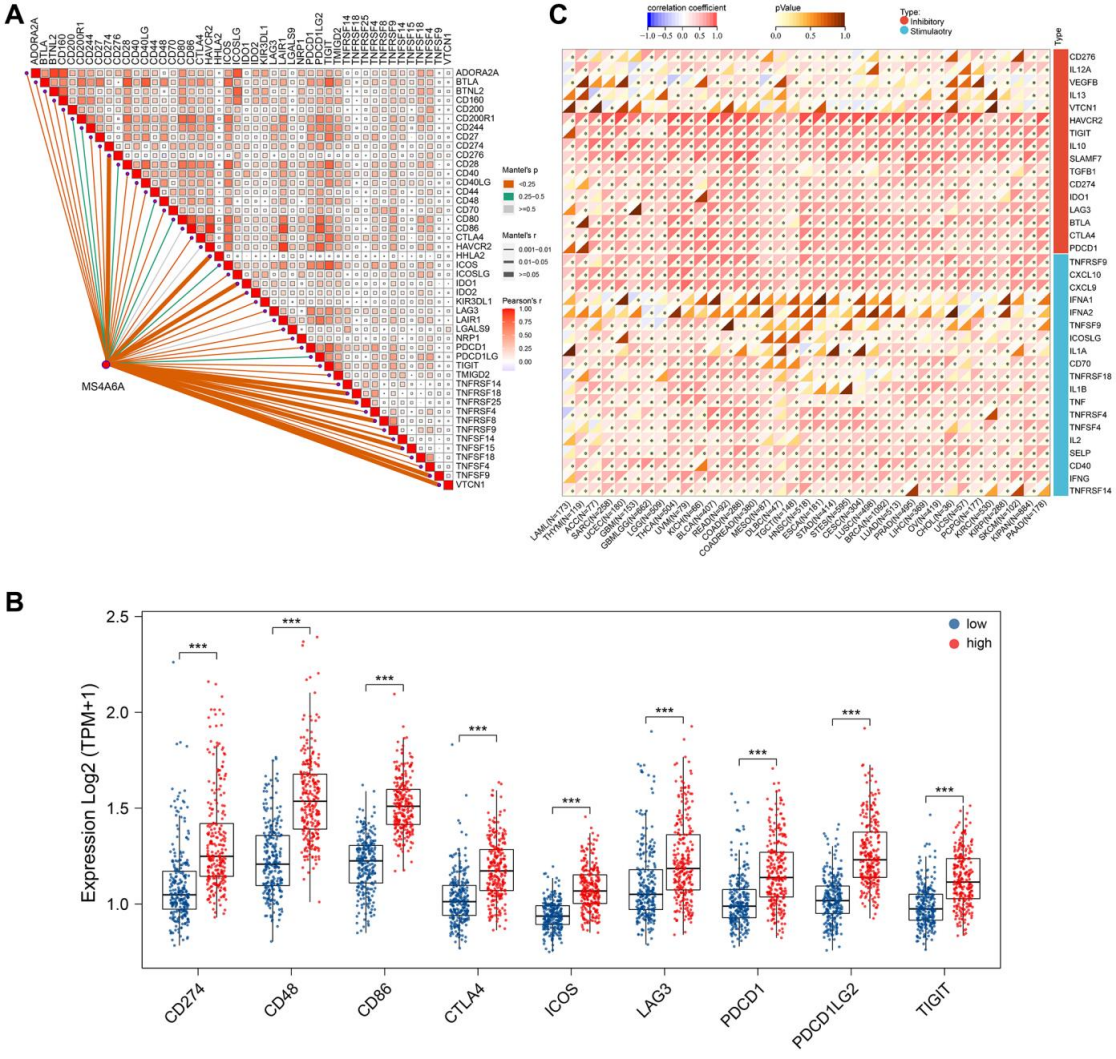
***MS4A6A* expression was positively correlated with ICP-related genes.** (**A**) Correlation of *MS4A6A* expression with ICP-related genes. (**B**) Comparison of *MS4A6A* expression with common immune checkpoint expression in TCGA-LUAD cohort. (**C**) Correlation of *MS4A6A* expression profile with ICP-related genes in the pan-cancer cohort. ^**^P < 0.01, ^***^P < 0.001.

Tumor cells activate immune checks so that antigens cannot be presented to T cells, thereby blocking the process of presenting antigens in the tumor immune link and suppressing the immune function of T cells. The use of anti-PD-1/PD-L1 in tumor treatment plays a significant role in immunotherapy [37]. We collected and analyzed 46 common genes associated with immune checkpoints to determine the relationship between *MS4A6A* and these genes [38]. Correlation analysis revealed that *MS4A6A* positively correlated with many immune checkpoint-associated genes (Figure 6A). We screened the most common immune checkpoint genes currently available, including PD-1 (*PDCD1*), PD-L1 (*CD274*), *CD48*, *CD86*, *CTLA4*, *ICOS*, *LAG3*, *PDCD1LG2*, and *TIGIT*, and compared the expression differences between the high and low *MS4A6A* expression groups. We found that all of them were highly expressed in the *MS4A6A* high expression group (Figure 6B). In addition, the four GEO datasets showed that immune checkpoint genes are generally elevated in the high-expression MS4A6A groups (Supplementary Figure 4). It is generally accepted that key regulators of immunity function in various tissues. Therefore, we investigated *MS4A6A* expression characteristics and immune checkpoint-associated gene expression in various cancer types. Notably, we observed that MS4A6A is positively correlated with genes associated with immune checkpoints in LUAD and 32 other cancer types (Figure 6C). These results suggest that *MS4A6A* expression may be associated with tumor immunotherapy response.

### MS4A6A can be used as a potential biomarker for immunotherapy

**Figure 7:**
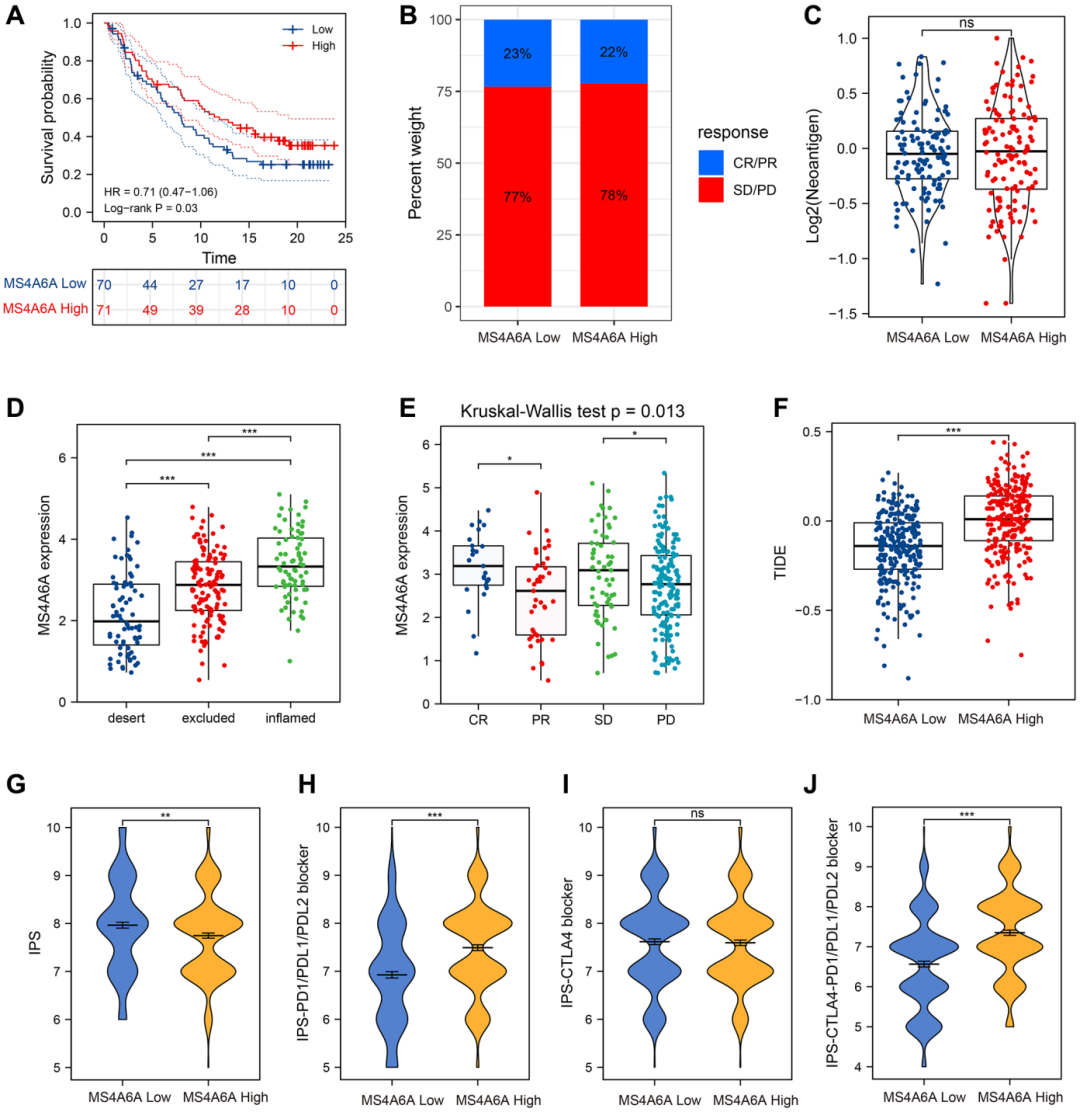
***MS4A6A* can be used as a potential biomarker for ICB treatment.** (**A**) OS curves for high and low *MS4A6A* expression in the IMvigor210 cohort. (**B**) The proportion of immune response in *MS4A6A* high and low expression groups against ICB treatment. Abbreviations: CR: complete remission; PR: local response; SD: stable disease; PD: progressive disease. (**C**) Comparison of neoantigen load between different *MS4A6A* expression subgroups. (**D**, **E**) Comparison of *MS4A6A* expression among different immune responsive cohorts. (**F**) TIDE differences in *MS4A6A* high- and low-expression cohorts. (**G**–**J**) TCIA analysis of differences in IPS scores in *MS4A6A* high and low expression cohorts. ^*^P < 0.05, ^**^P < 0.01, ^***^P < 0.001.

We then focused on immunotherapy-related datasets. To further confirm the predictive value of MS4A6A for immune checkpoint blockade (ICB) treatment, we collected transcriptomic profiles and clinical information from the uroepithelial carcinoma (UC) immunotherapy cohort (IMvigor210) [39]. Patients with high *MS4A6A* expression showed a more pronounced clinical benefit and significantly longer survival. (Figure 7A). In addition, patients with high *MS4A6A* expression had a significant treatment effect and immune response to PD-L1 blockade compared with patients with low *MS4A6A* gene expression (Figure 7B). When comparing neoantigen loads, no significant differences were found between the two groups with high or low *MS4A6A* expression (Figure 7C). However, patients with high *MS4A6A* expression had significantly higher tumor immunophenotypes and a better response to immunotherapy compared to those with lower expression (Figure 7D, 7E).

The TIDE and IPS scores have been widely used to predict the effects of immunotherapy. We compared the TIDE scores of patients with high and low *MS4A6A* expression in the TCGA-LUAD dataset. We observed that the TIDE scores of patients with high expression were significantly lower than those in the low expression group, suggesting that the effect of receiving ICB treatment might be better in patients in the high expression group (Figure 7F). TCIA results showed that the IPS was significantly higher in the low *MS4A6A* group (*P* < 0.001) (Figure 7G), and patients in the high *MS4A6A* expression group had a relatively higher response to anti-PD1/PDL1 treatment while there was no significant difference with anti-CTLA4 treatment (*P* < 0.001; Figure 7H–7J). These results suggest that patients with high MS4A6A expression may benefit from ICBs.

### Construction of a predictive model of immunotherapy based on *MS4A6A* and clinical features

**Figure 8:**
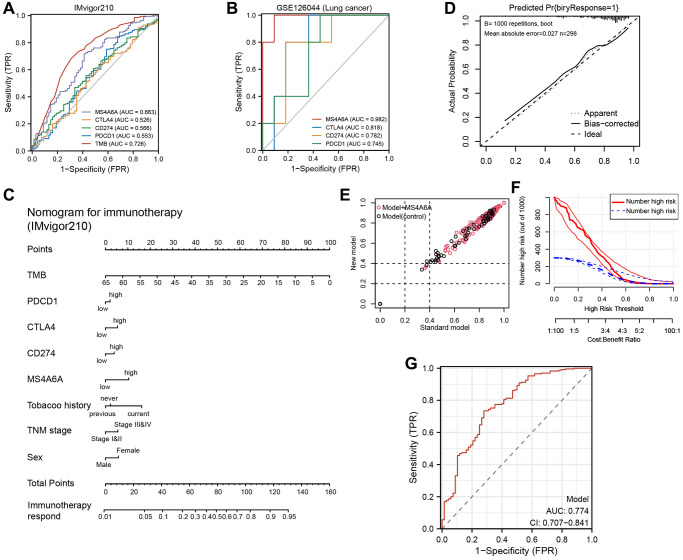
**Construction and validation of *MS4A6A*-based immunotherapy predictive model.** (**A**) ROC curves for *MS4A6A*, *PD-1*, *PD-L1*, and *CTLA4* based on the GSE126044 cohort. (**B**) ROC curves for TMB, *MS4A6A*, *PD-1*, *PD-L1*, and *CTLA4* based on the IMvigor210 cohort. (**C**) Immunotherapy prediction model nomogram. (**D**) Calibration curves of the immunotherapy prediction model. (**E**) Comparison of prediction accuracy of immunotherapy model with and without *MS4A6A*. (**F**) Clinical decision curves of the immunotherapy prediction model. (**G**) The ROC curve of clinical model (AUC = 0.774).

These results suggest that *MS4A6A* may be used as an immunotherapeutic marker; however, it is still unclear which markers are more effective than the expression of existing markers, such as *PD-1*, *PD-L1*, and *CTLA4*. Therefore, we plotted ROC curves based on the IMvigor210 cohort and found that the AUC values of *MS4A6A* (AUC = 0.663) were significantly higher than those of *PD-1* (AUC = 0.553), *CTLA4* (AUC = 0.526), and *PD-L1* (AUC = 0.566), but lower than that of TMB (AUC = 0.726) (Figure 8A); the accuracy of *MS4A6A* (AUC = 0.982) was better than that of *PD-1* (AUC = 0.745), *PD-L1* (AUC = 0.782), and *CTLA4* (AUC = 0.818) in the GSE126044 cohort (Figure 8B). Therefore, *MS4A6A* may be a better biomarker for immunotherapy than *PD-1*, *PD-L1*, or *CTLA4*. Subsequently, we investigated the MS4A6A protein expression in LUAD using the Human Protein Atlas (HPA) database. We observed that it was predominantly expressed in the nucleus and was significantly elevated in LUAD tissues compared to lung tissues. (Supplementary Figure 5).

Clinical prediction models are currently essential tools for aiding clinical decision-making. Here, a model that predicts immune therapy responses in patients using *MS4A6A* gene expression, as well as *PD-1*, *PD-L1*, and TMB, was developed to help clinicians make immunotherapy decisions. Using multivariate logistic regression, we constructed a prediction model for immunotherapy responders and non-responders (Figure 8C). Bootstrapping was used to assess the models, re-sampling 1000 times, and calibration curves were plotted (Figure 8D). The IDI scatter plot results showed that the accuracy of the prediction model, including *MS4A6A,* increased by 8% compared to that of the model without *MS4A6A* (Figure 8E). A DCA plot was used to reflect the net benefit of the patients from the models (Figure 8F). Then, the ROC curve of this model was plotted and the AUC value reached 0.774 (Figure 8G). Based on these results, *MS4A6A* combined with established immunotherapy markers can better predict immunotherapy outcomes.

## DISCUSSION

In recent years, immunotherapy for cancer has emerged as a remarkable advance in anti-cancer research, which has revolutionized cancer treatment and changed treatment strategies. In traditional cancer treatment procedures such as chemotherapy, cancer cells and stem cells are destroyed, causing irreversible damage and even death. For patients with advanced tumors, the advent of targeted therapies has brought new hope for longer survival. For instance, EGFR-tyrosine kinase inhibitors have significantly extended the survival time of many patients with advanced NSCLC [40]. Immunotherapy differs from conventional chemotherapy and targeted therapies in one essential way by targeting immune cells rather than cancer cells. Currently, for patients with advanced melanoma with cancer metastasis and for whom all treatment options have failed, Opdivo and Keytruda could reduce or even eliminate tumors in more than 60% of patients for more than two years [41–43]. The use of immunotherapy in treating lung cancer has shown remarkable results, greatly extending the survival time of patients with advanced lung cancer [2, 44–46]. Despite the correlation between immunotherapy responsiveness and certain immunomarkers, such as PD-1, PD-L1, and TMB, not all patients with high PD-1 or TMB expression benefit from immunotherapy. Single biomarkers have limitations that affect the accuracy of screening of populations that benefit from immunotherapy.

In the present study, we analyzed the characteristics of HRD and non-HRD populations of LUAD patients. Based on their differences in transcriptome levels, we screened a set of genes strongly associated with HRD. We screened independent prognostic factors by univariate Cox regression, LASSO, and multivariate Cox regression. Single-cell analysis was also carried out to explore the details of the independent prognostic factors at cell level. Notably, we found that *MS4A6A* gene expression was elevated in the HRD group and that patients with high *MS4A6A* expression had a better prognosis. *MS4A6A* has a higher expression in immune cells compared with other two cell types. This may suggest its association with tumor immune activity. MS4A family members play critical roles in various pathological conditions, including cancer, infectious diseases, and neurodegeneration. Also, they play a vital role in regulating immune signaling [47]. *MS4A6A*, also known as *CDA01*, *MS4A6*, *4SPAN3*, or *CD20L3*, encodes a member of the transmembrane 4A gene family. *MS4A6A* appears to be strongly associated with Alzheimer’s disease [48–50]; however, the *MS4A6A* gene has not been investigated. In the TCGA-LUAD dataset, we observed that *MS4A6A* expression was positively correlated with immune cell infiltration in the tumor microenvironment, especially macrophages, dendritic cells, and multiple immune checkpoints, suggesting that *MS4A6A* could be a potential biomarker for ICB therapy. By analyzing immunotherapy-related cohorts, we revealed that *MS4A6A* has a higher accuracy as a biomarker than molecules such as PD-1 and CTLA4. We constructed a predictive immunotherapy model based on the IMvigor210 dataset. The C-index and calibration curve results indicated that the model had good accuracy and consistency.

However, the present study has some limitations. Although there are some LUAD immunotherapy datasets in public databases, many are panel data instead of complete transcriptional data; thus, the expression data of *MS4A6A* were unavailable, and only GSE126044 was eligible for inclusion in this study. In addition, IMvigor210 cohorts were obtained from patients with bladder epithelial carcinoma, containing transcriptome data and comprehensive clinical information; therefore, it has been used in several immunotherapy-related and LUAD-related studies [51]. However, its application in LUAD studies remains controversial, considering the heterogeneity of tumors. Finally, the lack of wet-lab experiments using cell line models or human lung adenocarcinoma tumor tissues limits the confidence and applicability of our results, as it does not provide experimental evidence supporting the biological relevance and functional implications of the findings. In our future work, we will consider conducting wet-lab experiments to address the limitations of this study and complement our results.

## CONCLUSION

We identified *MS4A6A*, whose expression level was closely correlated with the level of HRD in LUAD and was highly accurate as an immunotherapeutic biomarker. Furthermore, detecting MS4A6A expression in tissues or blood is more straightforward than calculating the HRD scores. Nonetheless, its practicality must be confirmed in a larger cohort and prospective studies.

## Supplementary Materials

Supplementary Figures

Supplementary Table 1

Supplementary Table 2

Supplementary Table 3
