# Supplementary material for: Identification of immunotherapy biomarkers for improving the clinical outcome of homologous recombination deficiency patients with lung adenocarcinoma
Source: Aging (Albany NY). 2023 Aug 11;15(16):8090–112. doi: 10.18632/aging.204957 (PMC10496994; doi:10.18632/aging.204957)
Supplement: Supplementary Figures [file aging-15-204957-s001.pdf]

SUPPLEMENTARY FIGURES

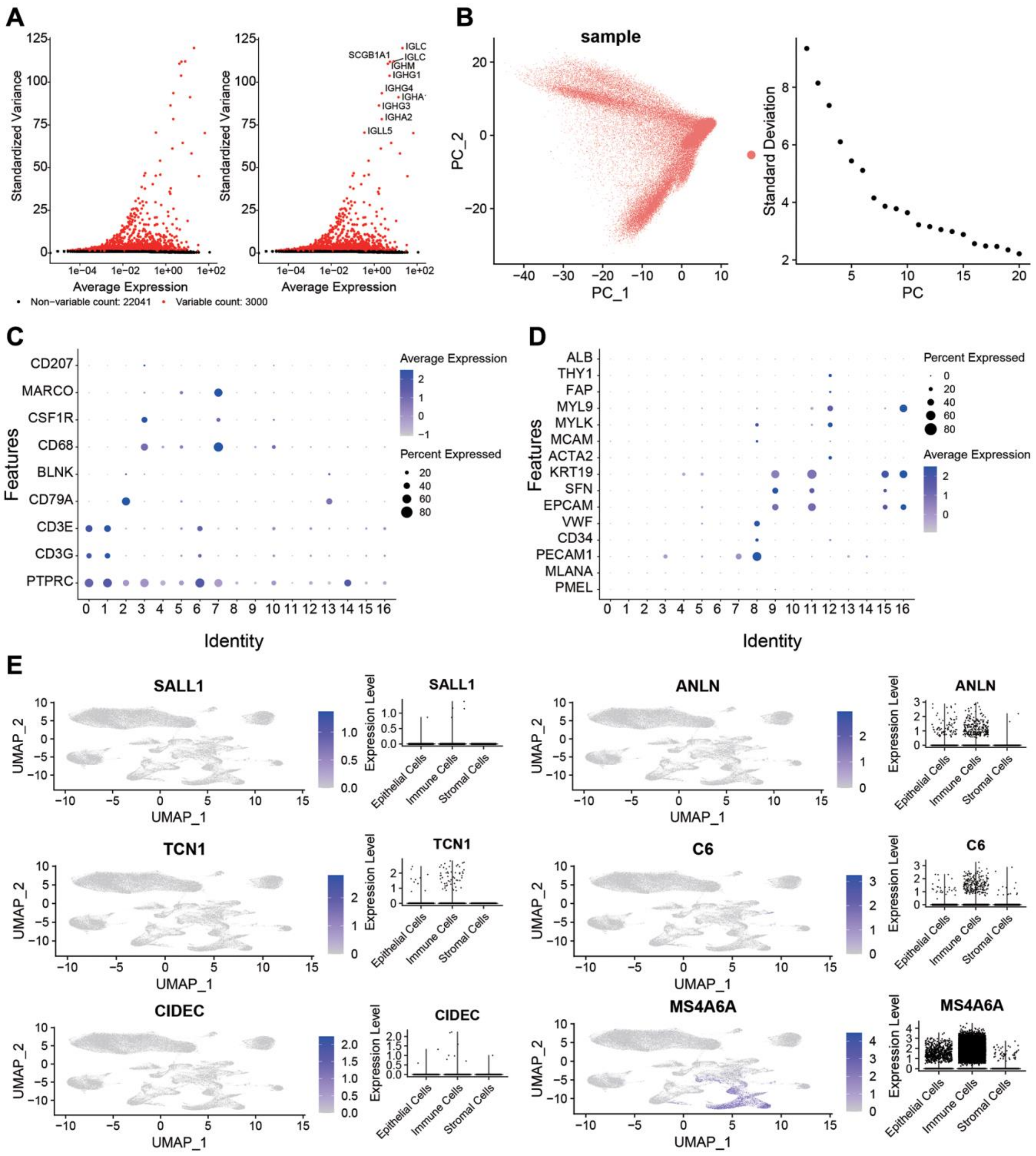

Supplementary Figure 1. scRNA-seq analysis. (**A**) The Variable Features plot identified the top 3000 variable genes, and the top 10 were highlighted. (**B**) PCA dimension reduction, 15 PCs were applied for cell clustering. (**C**) Bubble plot depicting the biomarkers of immune cells across 0–16 subsets. (**D**) Bubble plot depicting the biomarkers of epithelial and stromal cells across 0–16 subsets. (**E**) Distribution of 6 genes in different types of cells.

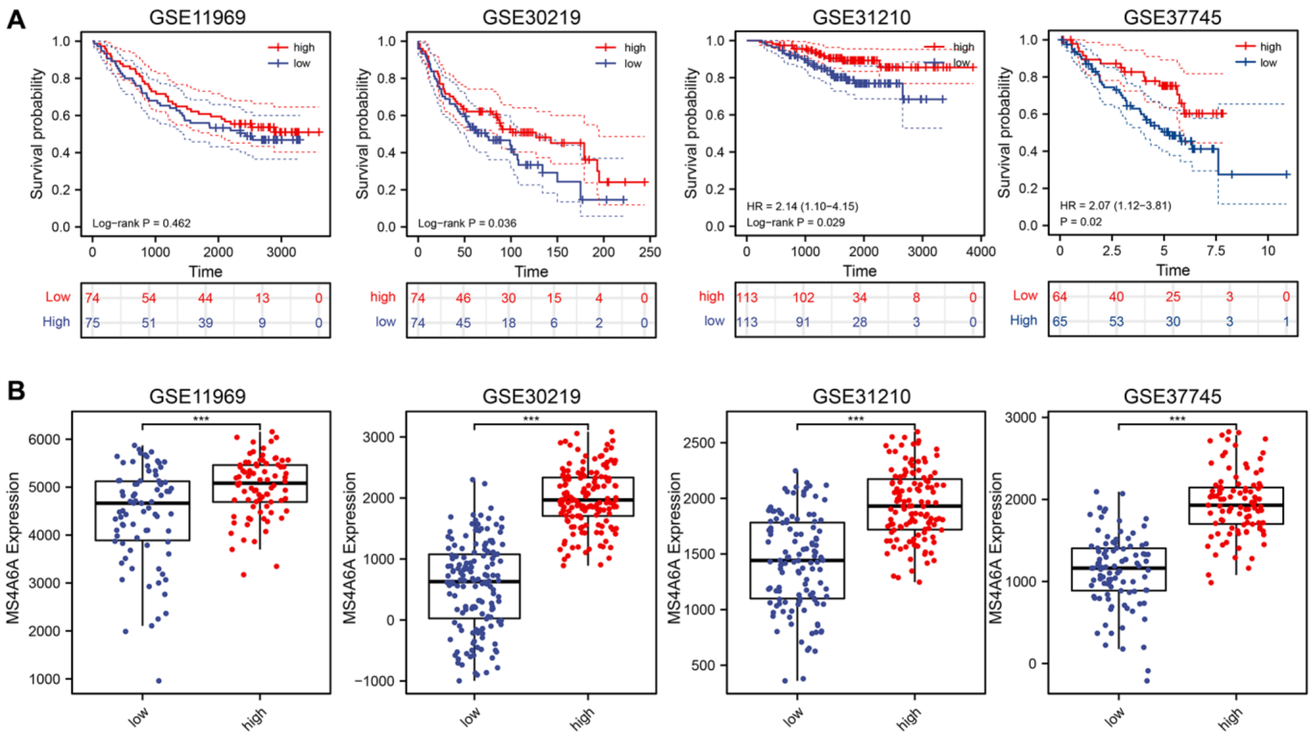

Supplementary Figure 2. Comparison of *MS4A6A* survival analysis and immune scores in GEO dataset. (**A**) KM survival curves of *MS4A6A* in the GSE11969, GSE30219, GSE31210, and GSE37745 datasets. (**B**) Differences in *MS4A6A* expression levels were compared in GSE11969, GSE30219, GSE31210, and GSE37745 in the high-low immune infiltration groups. *MS4A6A* expression was elevated in the high immune infiltration group.

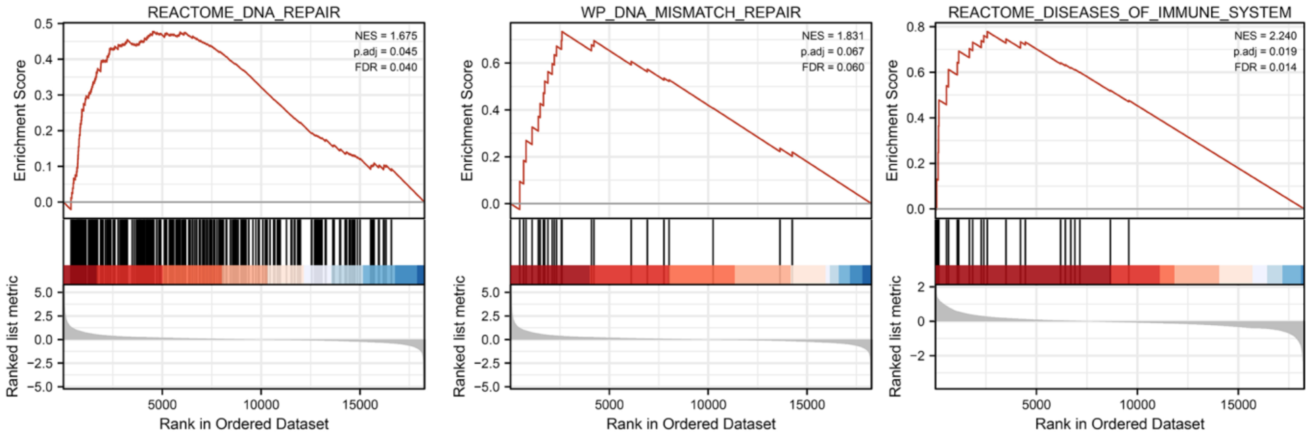

Supplementary Figure 3. GSEA of high and low expression patterns of *MS4A6A*. GSEA results revealed that the DNA repair, DNA mismatch repair, and immune system-related pathways were significantly enriched in the *MS4A6A*-positive group.

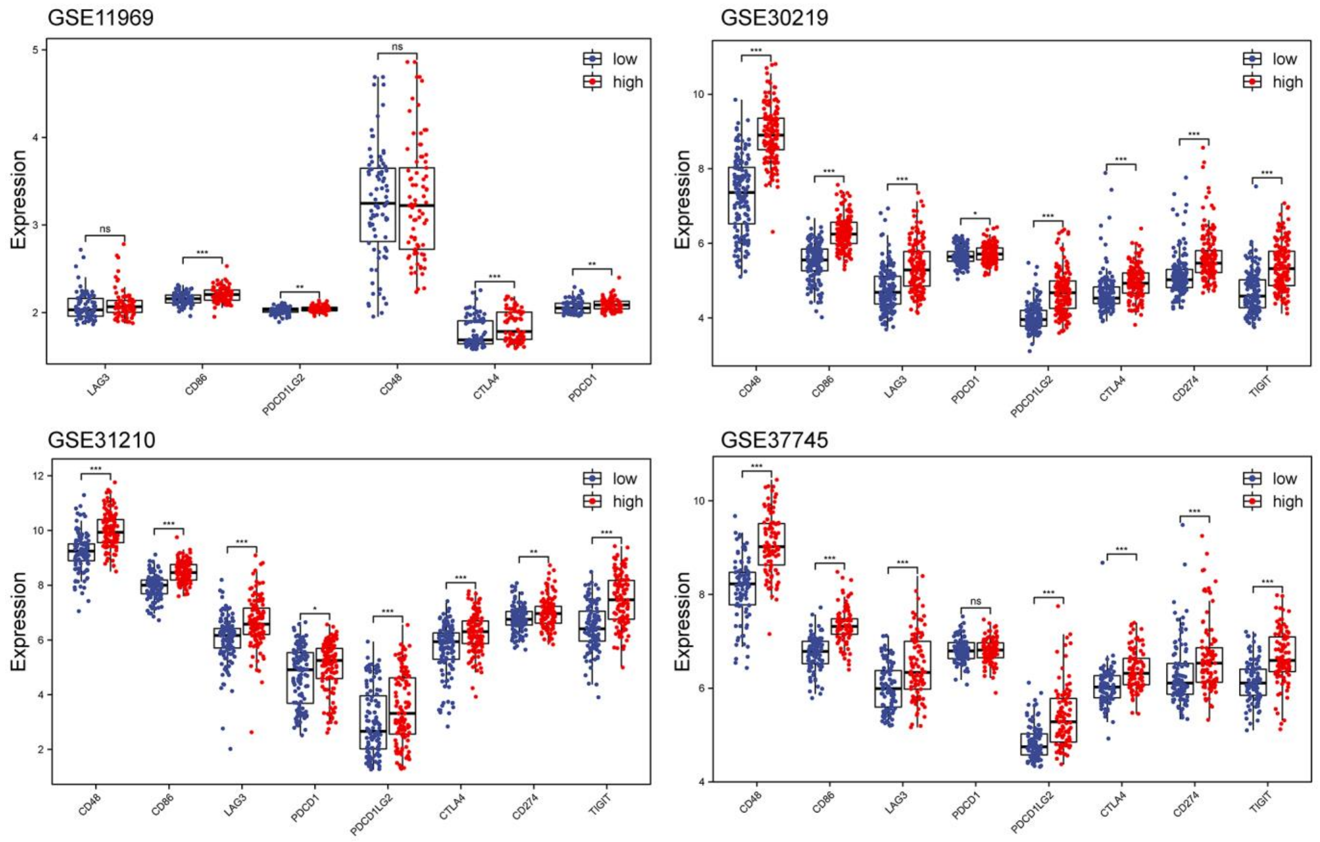

Supplementary Figure 4. Validation of relationship between *MS4A6A* gene and immune checkpoint expression in GEO dataset. Box plots demonstrate that *MS4A6A* high expression group in GSE11969, GSE30219, GSE31210, and GSE37745 datasets, as well as immune checkpoints, exhibited consistent elevated expression results with the TCGA-LUAD cohort.

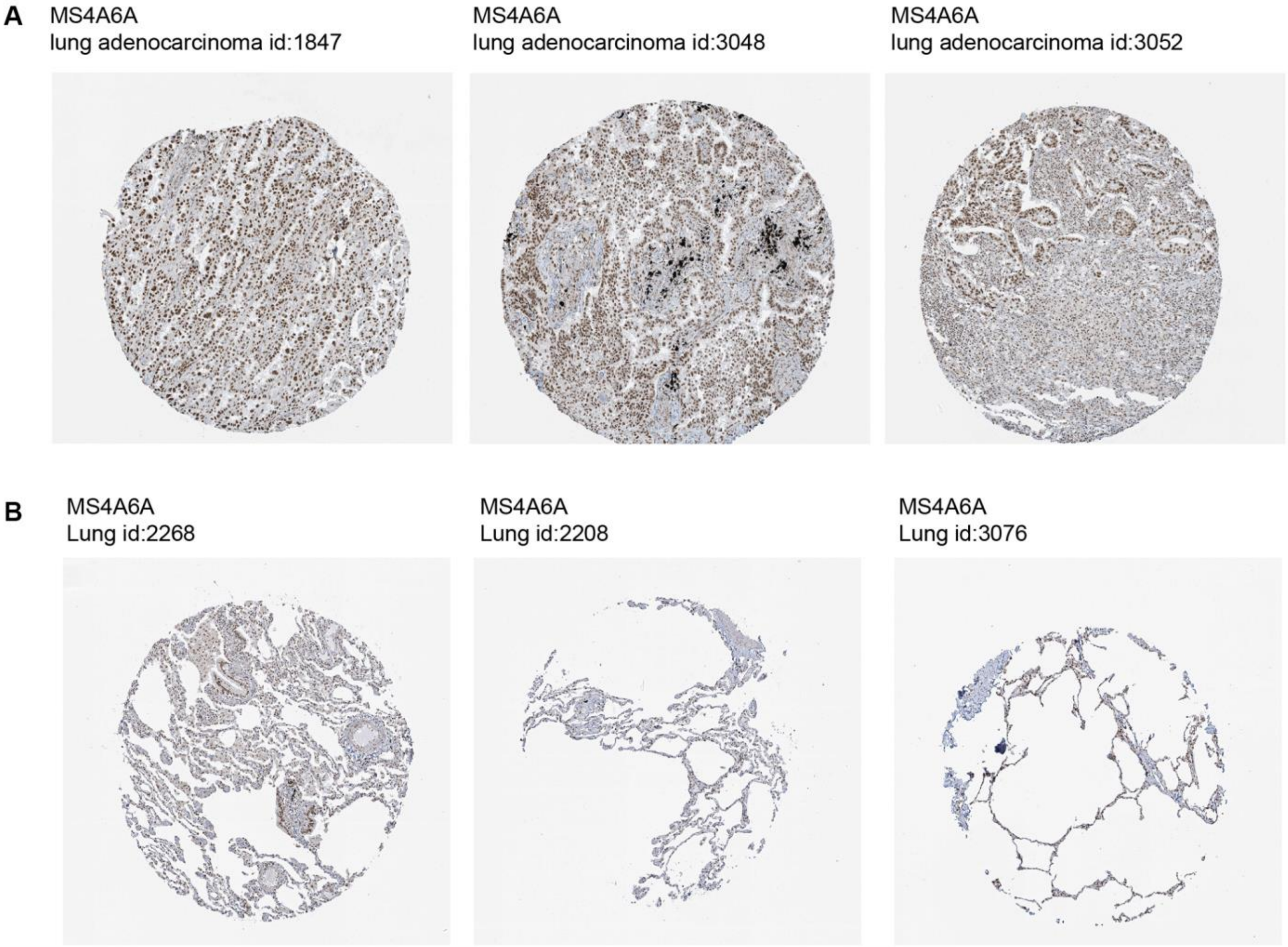

Supplementary Figure 5. Immunohistochemical analysis of MS4A6A in normal lung vs. LUAD tissues. (**A**) Immunohistochemistry of MS4A6A in LUAD tissue. (**B**) Immunohistochemistry of MS4A6A in normal lung tissue.
