# Supplementary material for: Identification of immunotherapy biomarkers for improving the clinical outcome of homologous recombination deficiency patients with lung adenocarcinoma
Source: Aging (Albany NY). 2023 Aug 11;15(16):8090–112. doi: 10.18632/aging.204957 (PMC10496994; doi:10.18632/aging.204957)
Supplement: Supplementary Table 1 [file aging-15-204957-s002.pdf]

## SUPPLEMENTARY TABLE

**Supplementary Table 1. Information about the GEO datasets employed.**

| | GSE11969 | GSE30219 | GSE31210 | GSE37745 | GSE189357 |
|---|---|---|---|---|---|
| Samples | 163 | 307 | 246 | 196 | 9 |
| Normal | 5 | 14 | 20 | 0 | 0 |
| Tumor | 158 | 293 | 226 | 196 | 9 |
| Platform | GPL7015 | GPL570 | GPL570 | GPL570 | GPL24676 |
| Data type | Expression profiling by array | Expression profiling by array | Expression profiling by array | | 10X genomics |
